# The requirement of Mettl3-promoted *MyoD* mRNA maintenance in proliferative myoblasts for skeletal muscle differentiation

**DOI:** 10.1098/rsob.170119

**Published:** 2017-09-06

**Authors:** Kensuke Kudou, Tetsuro Komatsu, Jumpei Nogami, Kazumitsu Maehara, Akihito Harada, Hiroshi Saeki, Eiji Oki, Yoshihiko Maehara, Yasuyuki Ohkawa

**Affiliations:** 1 Division of Transcriptomics, Medical Institute of Bioregulation, Kyushu University, JST-CREST, Fukuoka 812-8582, Japan; 2 Department of Surgery and Science, Graduate School of Medical Sciences, Kyushu University, Fukuoka 812-8582, Japan

**Keywords:** MyoD, Mettl3, HuR, m^6^A-sequencing, RNA processing, splicing

## Abstract

Myogenic progenitor/stem cells retain their skeletal muscle differentiation potential by maintaining myogenic transcription factors such as MyoD. However, the mechanism of how MyoD expression is maintained in proliferative progenitor cells has not been elucidated. Here, we found that *MyoD* expression was reduced at the mRNA level by cell cycle arrest in S and G2 phases, which in turn led to the absence of skeletal muscle differentiation. The reduction of *MyoD* mRNA was correlated with the reduced expression of factors regulating RNA metabolism, including methyltransferase like 3 (Mettl3), which induces N^6^-methyladenosine (m^6^A) modifications of RNA. Knockdown of Mettl3 revealed that *MyoD* RNA was specifically downregulated and that this was caused by a decrease in processed, but not unprocessed, mRNA. Potential m^6^A modification sites were profiled by m^6^A sequencing and identified within the 5′ untranslated region (UTR) of *MyoD* mRNA. Deletion of the 5′ UTR revealed that it has a role in *MyoD* mRNA processing. These data showed that Mettl3 is required for *MyoD* mRNA expression in proliferative myoblasts.

## 1. Introduction

Stem cells are able to self-renew and to multiply rapidly while maintaining their pluripotency, resulting in histogenesis [1,2]. The differentiation process from stem cells to various tissues has previously been elucidated by studies that characterized the factors or conditions required for differentiation into each organ or tissue [3–9]; by contrast, it is unclear how the differentiation potential is maintained in proliferative cells. In the case of skeletal muscle, which accounts for approximately 40% and 30% of adult male and female body weights, respectively [10], it is reasonable to assume that the differentiation of stem cells and progenitors requires multiple rounds of cell division to attain a high muscular density. However, the means by which the myogenic potential is maintained in proliferative skeletal muscle stem cells and progenitors remains to be determined.

The identification of MyoD and the MyoD family of myogenic regulatory factors (MRFs) has revealed that the myogenic potential is determined by the expression of transcription factors that influence myogenic gene expression. Conversely, the loss of MyoD and/or MRFs in skeletal myoblasts, both *in vitro* in C2C12 cells [11,12] and *in vivo*, suppresses skeletal myogenesis [13,14]. During differentiation, together with Pbx homeodomain protein, MyoD binds to the regulatory regions of myogenic genes, including *Myog*, *Mylpf*, *Myh3*, *Tnni* and *Ryr1* [15], which triggers chromatin remodelling by recruiting the SWI/SNF chromatin remodelling factor Brg1 for myogenic gene transcription [16,17]. Before differentiation, MyoD labels myogenic gene loci by incorporating the variant histone H3.3 with chromodomain helicase DNA-binding domain 2 (Chd2), without activating transcription [8]. Thus, during proliferative phases, MyoD is critical for myoblast differentiation and the inheritance of differentiation potency.

Transcription factor expression is destabilized by RNA modifications that influence the differentiation potential of stem cells [18,19]. In embryonic stem (ES) cells, methyltransferase like 3 (Mettl3) [20] induces an N^6^-methyladenosine (m^6^A) modification in *Nanog* RNA, which is required for pluripotency and differentiation [19]. m^6^A modifications have been reported to affect RNA function by various mechanisms, including splicing, stabilization/destabilization [18,21], nuclear export [22] and translation efficiency [23,24]. RNA modifications or stabilization may also be crucial for skeletal muscle differentiation because *MyoD* mRNA has a short half-life of approximately 90 min [25,26]. The RNA-binding protein HuR has been reported to stabilize *MyoD* mRNA and to be necessary for terminal skeletal muscle differentiation [25,27]. It was also shown that *MyoD* mRNA levels are quite low in G0-arrested cells but increase upon re-entry into the cell cycle [28], implying that *MyoD* mRNA could be stabilized during the cell cycle. However, the underlying mechanisms, as well as the factors required for the maintenance of MyoD expression during proliferation, remain to be clarified.

Here, we explored the maintenance of *MyoD* mRNA levels in proliferative myoblasts. We found that cell cycle arrest reduced *MyoD* mRNA expression, thus suppressing myogenic differentiation, and that Mettl3 stabilized *MyoD* mRNA by promoting mRNA processing in skeletal myoblasts. Our results suggest that m^6^A modification by Mettl3 stabilizes *MyoD* mRNA levels for skeletal muscle differentiation.

## 2. Results

### 2.1. Cell cycle arrest in S and G2 phases reduces *MyoD* mRNA levels and inhibits myoblast differentiation

**Figure 1.**
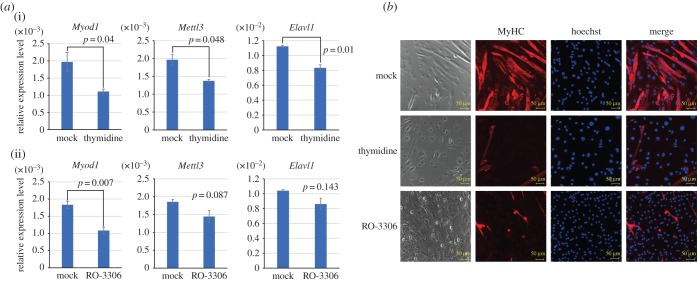
*MyoD* mRNA expression is reduced upon cell cycle arrest. (*a*) mRNA levels of *Myod1*, *Mettl3* and *Elavl1* in C2C12 cells treated with thymidine or RO-3306 for 48 h. (i) Cells treated with thymidine were compared with non-treated cells. (ii) Cells treated with RO-3306 were compared with DMSO-treated cells. (*b*) Morphology of C2C12 cells treated with DMSO (labelled as Mock), thymidine and RO-3306. RO-3306 was used as a Cdk1 inhibitor. Cells were immunostained for anti-MyHC and Hoechst at 72 h after the induction of differentiation. MyHC, myosin heavy chain.

During proliferative phases, MyoD binding to target genes such as myogenic genes is required for skeletal muscle differentiation [8,13,29], suggesting that the maintenance of MyoD expression during cell cycle progression could be critical for differentiation. Because *MyoD* mRNA levels were reported to be low following cell cycle arrest at G0 [28], we hypothesized that cell cycle arrest may cause *MyoD* mRNA instability. C2C12 cells, a mouse myoblast cell line with both self-renewal and differentiation potential, were arrested either in the S phase by thymidine or in the G2 phase by the Cdk1 inhibitor RO-3306, and *MyoD* mRNA levels were analysed by quantitative reverse transcription PCR (qRT-PCR). Cell cycle arrest was confirmed by measuring the population in each cell cycle phase after exposure to thymidine or RO-3306 (electronic supplementary material, figure S1*a*). We observed cell cycle re-entry and the proliferation of cells after removal of the inhibitors (electronic supplementary material, figure S1*b*), confirming that the drug treatments did not induce cell death under our experimental conditions. qRT-PCR analysis showed that *MyoD* mRNA levels were significantly reduced after cell cycle arrest in both S and G2 phases in the growth state (*p* = 0.04 and 0.007, respectively; figure 1*a*(i)). On the other hand, mRNA levels of other skeletal muscle-specific transcription factors (*Pax7* and *Srf*) were not substantially affected by cell cycle arrest in the proliferative state (electronic supplementary material, figure S2*a*).

Next, we examined the skeletal muscle differentiation of arrested C2C12 cells. Cells were cultured with 2% horse serum (HS) media for 48 h to induce differentiation. We found that myotube formation was diminished in thymidine- and RO-3306-treated cells but not in mock-treated cells (figure 1*b*). qRT-PCR analysis revealed that mRNA levels of *MyoD* as well as those of the skeletal muscle-specific genes *Myog* and *Acta1* were decreased by cell cycle arrest in the differentiated state (electronic supplementary material, figure S2*b*). Taken together, these results suggest that during proliferative phases, cell cycle arrest leads to a reduction of *MyoD* mRNA expression that is required for skeletal muscle differentiation.

### 2.2. Cell cycle arrest affects mRNA levels of *HuR* and *Mettl3*

To examine the mechanism by which *MyoD* mRNA expression is decreased upon cell cycle arrest, we focused on two pathways potentially involved in RNA metabolism. One was the HuR (also known as Elavl1)-mediated stabilization of RNA, which involves HuR binding to AU-rich elements of *MyoD* mRNA in the early stages of skeletal myoblast differentiation [25,27,30]. The other was the m^6^A modification of RNA introduced by Mettl3 [20], which is also important for RNA stabilization. qRT-PCR analysis showed that *Elavl1* and *Mettl3* mRNA levels were significantly reduced by cell cycle arrest using thymidine (both *p* < 0.05; figure 1*a*(i)), but the effect was relatively limited following treatment with RO-3306 (*p* = 0.087 and 0.143, respectively; figure 1*a*(ii)). These results suggest that the decreased expression of HuR and/or Mettl3 may account for the decline in *MyoD* mRNA expression upon cell cycle arrest.

### 2.3. Knockdown of Mettl3, but not of HuR, downregulates *MyoD* mRNA levels in skeletal myoblasts

**Figure 2.**
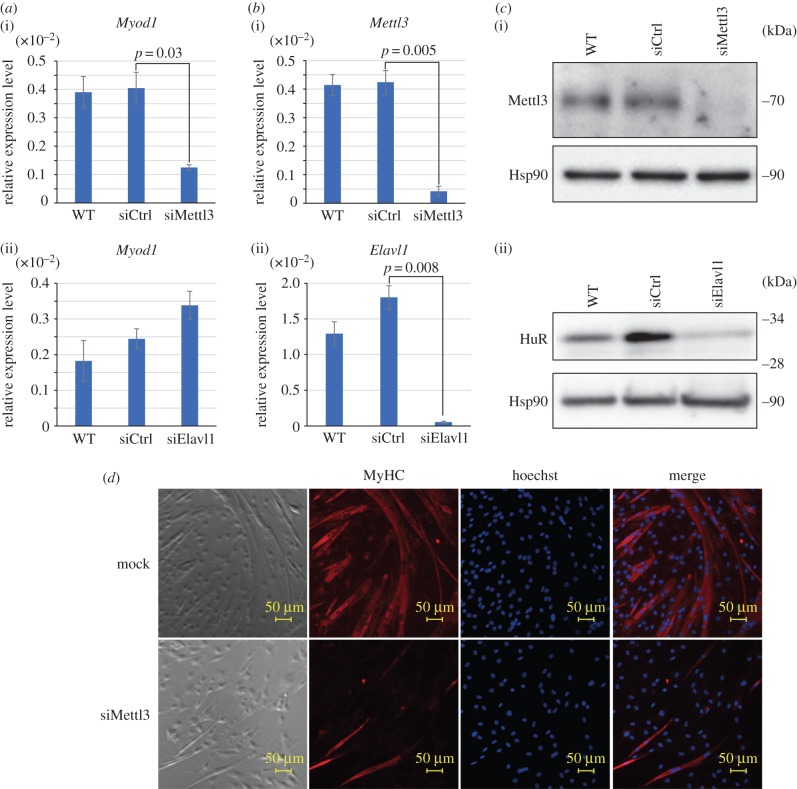
Knockdown of Mettl3, but not HuR, downregulates *MyoD* mRNA levels and suppresses skeletal muscle differentiation. (*a*) qRT-PCR analysis of *Myod1* in (i) Mettl3 and (ii) HuR knockdown cells. (*b*) qRT-PCR analysis to monitor (i) Mettl3 and (ii) HuR knockdown efficiency. (*c*) Western blot analysis to monitor (i) Mettl3 and (ii) HuR knockdown efficiency. Hsp90 was used as a loading control. (*d*) Morphology of C2C12 cells with transfected Mettl3 siRNA. Cells were immunostained for anti-MyHC and Hoechst at 72 h after the induction of differentiation.

Next, to determine if HuR and/or Mettl3 regulate *MyoD* mRNA expression, we carried out small interfering (si)RNA-mediated knockdown of Mettl3 and HuR. qRT-PCR showed that *MyoD* mRNA levels were significantly reduced in Mettl3 knockdown cells (*p* = 0.03; figure 2*a*(i)), but not in HuR knockdown cells (figure 2*a*(ii)). Both siRNAs suppressed the expression of target genes at the mRNA (figure 2*b*) as well as protein level (figure 2*c*), validating the knockdown efficiency. These data suggest that Mettl3, but not HuR, is implicated in maintaining *MyoD* mRNA levels. Mettl3-targeted siRNA treatment also suppressed myotube formation (figure 2*d*).

### 2.4. Knockdown of Mettl3 markedly downregulates processed, but not unprocessed, *MyoD* mRNA levels

**Figure 3.**
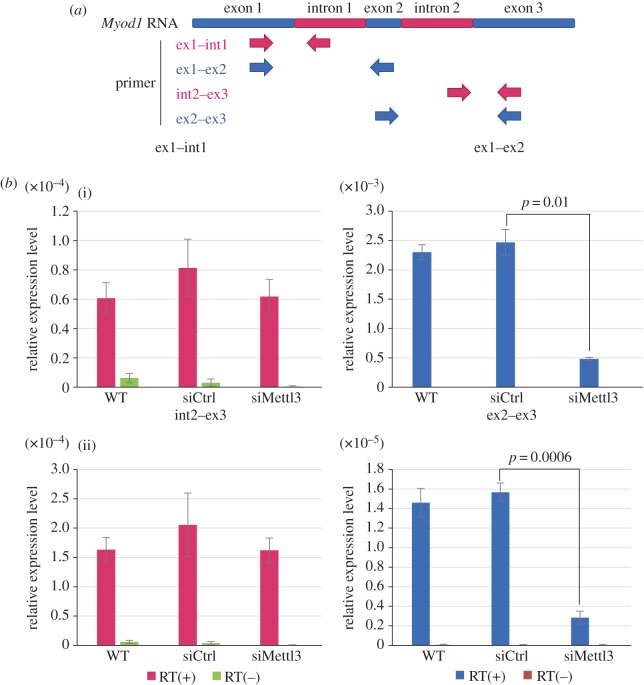
Knockdown of Mettl3 downregulates processed, but not unprocessed, *MyoD* mRNA levels. (*a*) Schematic of *Myod1* RNA which consists of three exons and two introns. Four primer pairs were designed: one with a forward primer in exon 1 and a reverse primer in intron 1, one with a forward primer in exon 1 and a reverse primer in exon 2, one with a forward primer in intron 2 and a reverse primer in exon 3, and one with a forward primer in exon 2 and a reverse primer in exon 3. ex: exon, int: intron. (*b*) qRT-PCR analysis of *Myod1* in Mettl3 knockdown C2C12 cells using the above four primer pairs. To confirm the absence of DNA contamination, each sample was also analysed without reverse transcriptase. RT: reverse transcriptase. Error bars show ± s.d. (*n* = 3). *p*-value versus control siRNA.

Because m^6^A modification by Mettl3 has been proposed to regulate RNA metabolism such as splicing [31–35], we examined the processing of *MyoD* pre-mRNA for the generation of mature *MyoD* mRNA. To investigate whether Mettl3 affects *MyoD* RNA splicing, two primer pairs were designed to anneal between two of the three *MyoD* exons and another two pairs to include both an intron and an exon (figure 3*a*). qRT-PCR analysis showed that in Mettl3 knockdown cells, fewer PCR products containing only exons were obtained (figure 3*b*(ii)), whereas there was no effect on the amount of PCR products containing an intron (figure 3*b*(i)). The length of PCR products amplified with exonic primer pairs was the correct splice size, and non-spliced RNA was not amplified (electronic supplementary material, figure S3*a*). Taken together, this indicates that knockdown of Mettl3 significantly decreased the amount of processed *MyoD* mRNA without affecting that of unprocessed *MyoD* RNA.

To investigate whether the m^6^A reading process is associated with the maintenance of *Myod1* mRNA, we focused on the representative m^6^A readers YTHDF2, YTHDC1, HNRNPA2B1 and HNRNPC [18,24,31–36], which were previously shown to be involved in RNA stability and processing [34,35]. We performed siRNA-mediated knockdown of these four factors and found that *MyoD* mRNA expression and myotube formation were only suppressed by Ythdf2 knockdown (electronic supplementary material, figure S3*b*,*c*). Knockdown of Ythdf2, however, led to a decrease of both processed and unprocessed *MyoD* RNA (electronic supplementary material, figure S3*d*), unlike the case of Mettl3 knockdown.

### 2.5. m^6^A modifications of *MyoD* mRNA are primarily enriched in the 5′ untranslated region

**Figure 4.**
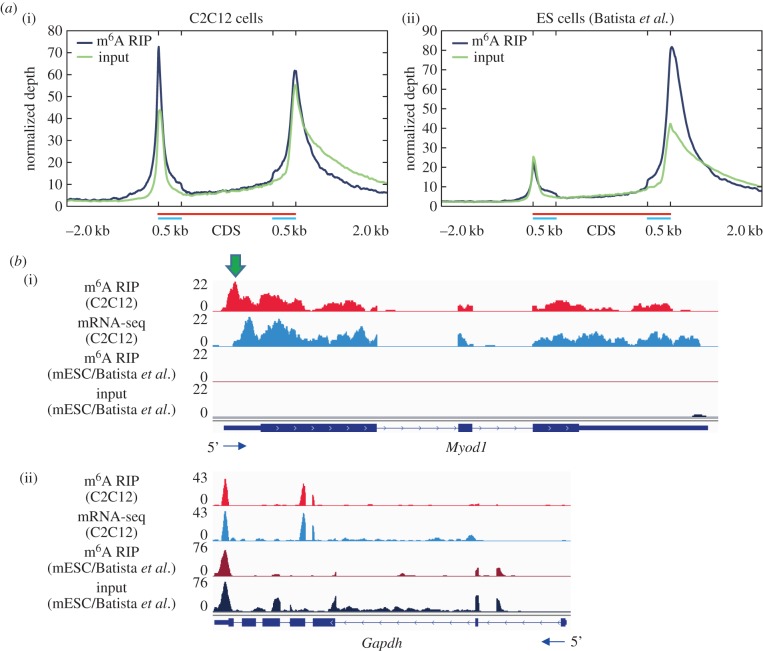
m^6^A modifications on *MyoD* mRNA are primarily found in the 5′ UTR. (*a*) Distribution of m^6^A signals across all mRNAs in (i) C2C12 cells and (ii) mouse ES cells. The lengths of all coding sequences (CDS) were scaled to 2 kb for visualization (indicated by red bars), except for 500 bp at both ends (blue bars). (*b*) Integrative Genomics Viewer tracks displaying reads coverage of (i) *Myod1* and (ii) *Gapdh* in m^6^A-seq and mRNA-seq data for C2C12 cells and mouse embryonic stem cells (mESC) (SRR1207291 (input), SRR1207292 (m^6^A RIP), Batista *et al*. [19]).

To further investigate the m^6^A modification of *Myod1* mRNA, m^6^A-sequencing (m^6^A-seq) was conducted by pulling down transcripts with an antibody that specifically recognizes m^6^A modifications [31]. Published studies have reported that m^6^A modifications are distributed in 3′ UTRs, stop codons and internal long exons, but rarely in 5′ UTRs [19,31,37,38] (figure 4*a*(ii)), while we observed enriched m^6^A signals in C2C12 cells both near the start codon and stop codon (figure 4*a*(i)). Integrative Genomics Viewer plots showed that while m^6^A modifications on the majority of RNAs such as *Gapdh* and *Srf* were enriched around 3′ UTRs, stop codons and long exons (figure 4*b*; electronic supplementary material, figure S4), m^6^A modifications of *Myod1* were notably enriched around the 5′ UTR (figure 4*b*(i), green arrow).

We also found that C2C12 cells and ES cells had many analogous loci for m^6^A signal enrichment (figure 4*b*(ii); electronic supplementary material, figure S4, upper panel). However, several skeletal muscle-specific genes such as *Myod1* and *Myog* were enriched in m^6^A signals only in C2C12 cells, whereas m^6^A signal enrichment in pluripotent genes such as *Nanog* was observed only in ES cells (figure 4*b*(i); electronic supplementary material, figure S4, middle and lower panels), reflecting cell type-specific regulations.

### 2.6. *Myod1* 5′ UTR is required for the maintenance of processed *Myod1* mRNA during cell proliferation

**Figure 5.**
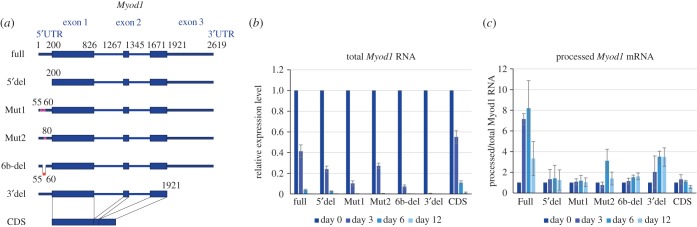
Conservation of the *Myod1* 5′ UTR stabilizes processed *Myod1* mRNA. (*a*) Schematic of *Myod1* mutants. ‘Full’ consists of full-length *Myod1* DNA. ‘5'del’ and ‘3'del’ are mutants with deleted 5′ UTRs and 3′ UTRs, respectively. In ‘Mut1,’ both 55A and 60A were replaced with C. In ‘Mut2,’ 80A was replaced with C. ‘6b-del’ carries a 55A to 60A deletion. ‘CDS’ contains only *Myod1* CDS (i.e. the 5′ UTR, 3′ UTR, and all introns were deleted). (*b*) qRT-PCR analysis of total *Myod1* RNA with seven *Myod1* mutants. NIH3T3 cells were transfected with vectors shown in (*a*), and total *Myod1* RNA levels were analysed at days 0, 3, 6 and 12 using a primer set within exon 1. Data are represented as the mean ± s.d. (*n* = 3). (*c*) Accumulation levels of processed *Myod1* mRNA. Processed *Myod1* mRNA levels were analysed using a primer pair between exon 1 and exon 2. Processed *Myod1* mRNA was normalized to total *Myod1* RNA. Data are represented as the mean ± s.d. (*n* = 3).

To investigate the function of m^6^A modification on *MyoD* mRNA, especially around the 5′ UTR, we constructed vectors harbouring *Myod1* with various mutations and deletions in the 5′ UTR. ‘RRAC’ has been shown to be a common sequence of m^6^A sites, with ‘GGAC’ the most frequent of these [18,24,31,37,38]. m^6^A-seq revealed the presence of some ‘GGAC’ motifs within m^6^A modifications on *Myod1* 5′ UTR. Therefore, deleted or mutated versions of three ‘GGAC’ motifs in the *Myod1* 5′ UTR were generated, as well as an entire 5′ UTR deletion mutant, and an entire 3′ UTR deletion mutant (figure 5*a*). These mutants were transiently transfected into NIH3T3 cells, and qRT-PCR detected total (both processed and unprocessed) and processed *MyoD* RNA using primer pairs within exon 1 of *Myod1* and between exon 1 and exon 2, respectively.

The ectopic introduction of each vector could be monitored by total *MyoD* RNA levels, all of which led to a similar pattern of decline (figure 5*b*). To quantify the accumulation of processed *Myod1* mRNA, these levels were normalized against those of total *Myod1* RNA (figure 5*c*). When transfecting vectors containing full-length *Myod1* and 3′ UTR deletion mutants, which retained the complete 5′ UTR, we observed an accumulation of processed *Myod1* mRNA until day 6 (figure 5*c*, Full and 3′ del). By contrast, all 5′ UTR deletion mutants showed no significant accumulation of processed mRNA upon transfection (figure 5*c*, 5′del, Mut1, Mut2 and 6b-del). Collectively, these data suggest that *MyoD* RNA is actively stabilized by its 5′ UTR through the efficient processing of *Myod1* mRNA during cell proliferation.

## 3. Discussion

In this study, we showed that *MyoD* mRNA expression reduces upon cell cycle arrest. We also found that Mettl3 regulates the maintenance of *MyoD* mRNA levels through m^6^A modification of the 5′ UTR during cell proliferation. Taken together, these results suggest that the myogenic potential is maintained throughout the cell cycle, at least in part by the Mettl3-mediated stabilization of processed *MyoD* mRNA during proliferative phases.

m^6^A-seq data showed that *MyoD* mRNA contains m^6^A modifications, mainly in its 5′ UTR, while previous studies reported that m^6^A is more commonly seen in 3′ UTRs, stop codons and internal long exons (figure 4*a*(ii)) [19,31,37–39]. The function of m^6^A is largely dependent on modifications around the 3′ UTR; for example, YTHDF2 typically targets the stop codon region (42%), the coding region (36%) and the 3′ UTR (14%), while the proportion of all YTHDF2-binding sites in the 5′ UTR is only 2% [18]. However, a recent study found that a 5′ UTR m^6^A promoted cap-independent translation [23]. These lines of evidence suggest that the functions of m^6^A modification may differ between 5′ UTRs and 3′ UTRs.

Because Mettl3 knockdown in this study caused the immature processing of *MyoD* RNA, we evaluated the involvement of YTHDC1, which is a nuclear m^6^A reader that regulates mRNA splicing by recruiting splicing factors [32,33]. Our results showed, however, that knockdown of YTHDC1 did not change MyoD RNA level significantly (electronic supplementary material, figure S3*b*). Previous studies reported that target RNAs for YTHDF2 are destabilized by m^6^A modification and stabilized upon Mettl3 knockdown [18,19]. Our results, however, showed the opposite effects, and thus suggest that *MyoD* RNAs are unlikely to be a direct target of YTHDF2. This raises the possibility that YTHDF2 functions to indirectly stabilize *MyoD* mRNA through its many (greater than 3000) targets [18]. Future work should attempt to uncover the mechanisms involving these factors.

Although *HuR* and *Mettl3* mRNA expression was reduced by thymidine-mediated cell cycle arrest, this decrease was non-significant following treatment with RO-3306. Cell cycle arrest was planned for the standard duration of 48 h. While a single treatment of thymidine was adequate to achieve cell cycle arrest because its effects increase gradually for at least 48 h, RO-3306 acts rapidly within 8 h, but its effects are diminished by 12 h such that additional treatments were required at intervals of 8–12 h. Between additional doses, reactivated cells may have affected mRNA levels, which may explain the more prominent effects of thymidine compared with RO-3306. To directly examine the involvement of HuR and Mettl3 in the regulation of *MyoD* mRNA expression, we conducted siRNA-mediated knockdown experiments and found that knockdown of Mettl3, but not of HuR, significantly affected *MyoD* mRNA expression. Previous studies reported that HuR stabilizes *MyoD* mRNA by binding to its AU-rich elements during the early stages of differentiation [25]. The discrepancy between these results and our own remains to be resolved. Because our investigation was conducted during the growth state, HuR may have had little effect on *MyoD* mRNA expression before the onset of differentiation. Interestingly, it was previously reported that HuR is significantly associated with m^6^A bait [31], but the exact relationship remains to be elucidated.

In summary, *MyoD* mRNA levels appear to be maintained by Mettl3-mediated m^6^A modifications, suggesting that Mettl3 is a critical regulator for skeletal muscle differentiation. These findings help understand the mechanisms underlying the maintenance of myogenic potential in proliferative skeletal muscle progenitors. Moreover, the methods developed in this study may be beneficial in evaluating different cell types from various species.

## 4. Material and methods

### 4.1. Cell culture and drug treatment

The mouse myoblast cell line C2C12 was maintained in a growth state in Dulbecco's modified Eagle's medium (DMEM) (Gibco by Thermo Fisher Scientific) containing 1 g l^−1^ glucose, 1% l-glutamine and 1% penicillin/streptomycin supplemented with 20% fetal bovine serum (FBS). NIH3T3 cells were maintained in DMEM containing 4.5 g l^−1^ glucose, 1% l-glutamine and 1% penicillin/streptomycin supplemented with 20% FBS. Cells were grown in a 5% CO_2_ cell culture incubator at 37°C. Differentiation stimuli were induced by exchanging media with 2% HS instead of 20% FBS.

C2C12 myoblasts were arrested in S phase by thymidine (Sigma-Aldrich, T1895) and in G2 phase by Cdk1 inhibitor (RO-3306, Sigma-Aldrich, SML0569) for 48 h. A single treatment of thymidine was applied, while additional RO-3306 was added at intervals of 8–12 h.

### 4.2. Immunocytochemistry

Cells were cultured on µ-Plate 24 Well (ibidi), washed twice with phosphate-buffered saline (PBS), fixed with 1% paraformaldehyde in PBS, permeabilized with 0.5% Triton X-100 in PBS and washed twice with PBS. A 15 min incubation with Blocking One (Nacalai Tesque Inc.) was followed by a 2 h incubation with mouse anti-myosin heavy chain (MF20, eBioscience, 1 : 200) diluted with 10% Blocking One in PBS at room temperature. The µ-Plate 24 Well was then washed three times with PBS and incubated for 30 min at room temperature with CF568-labelled goat anti-mouse antibody (1 : 1000; Biotium Inc.) and Bisbenzimide H33342 Fluorochrome Trihydrochloride (Hoechst) (1 : 2000; Nacalai Tesque Inc.) diluted with 10% Blocking One in PBS. The µ-Plate 24 Well was again washed three times in PBS and mounted in ibidi Mounting Medium (ibidi). Images were visualized using a fluorescence microscope (BZ-9000; Keyence). Co-localization was evaluated using BZ-II Analyzer software (Keyence).

### 4.3. Fluorescence activated cell sorting

C2C12 cells in a growth state were isolated by trypsin treatment and centrifuged for 5 min at 190*g*. The cell pellet was resuspended in 1 ml of PBS supplemented with Hoechst 33342 stain (Nacalai Tesque Inc.) and incubated for 15 min. This suspension was analysed by a cell sorter (SONY, SH800).

### 4.4. Quantitative RT-PCR

Total RNA was extracted from cells using Sepasol-RNA I Super G (Nacalai Tesque Inc.) and ethanol precipitation. Total RNA (1 µg) was used for reverse transcription with the PrimeScript RT reagent kit (Takara Bio Inc., RR047A). qRT-PCR was performed using Thunderbird SYBR qPCR Mix (Toyobo Co., Ltd.) with the PikoReal 96 Real-Time PCR System (Thermo Fisher Scientific) as previously described [40]. Primers are listed in the electronic supplementary material, table S1. qRT-PCR data were normalized to *Gapdh* or *Eef1a1* expression levels and presented as the mean ± s.d. of three independent experiments. For qRT-PCR of processed *MyoD* mRNA in NIH3T3 cells with transfected MyoD mutants, differences in transfection efficiencies were normalized by using total *MyoD* RNA in each sample as an internal control. Total *MyoD* RNA levels were quantified by performing qRT-PCR using primer pairs within exon 1 of *Myod1*, while processed *MyoD* mRNA was quantified using primer pairs between exon 1 and exon 2.

### 4.5. Western blotting

Cells were harvested and disrupted in a Sample Buffer Solution with 2-ME (Nacalai Tesque Inc.). Samples were separated by sodium dodecyl sulfate polyacrylamide gel electrophoresis using a 5–20% Extra PAGE One Precast Gel (Nacalai Tesque Inc.) and electrotransferred to a polyvinylidene fluoride membrane with the Trans-Blot Turbo Transfer System (Bio-Rad Laboratories, Hercules, CA; 2.5 A, 25 V, 7 min). The membrane was blocked for 1 h in 5% (w/v) skimmed milk in Tris-buffered saline containing 0.05% (v/v) Tween 20, then incubated with primary antibodies in Hikari Solution A (Nacalai Tesque Inc.), followed by incubation with secondary antibodies and detection using Chemi-Lumi One Ultra (Nacalai Tesque Inc.).

The following primary antibodies were used for western blotting: rat anti-MyoD (5F11, Millipore 1 : 500), anti-Mettl3 (15073-1-AP, Proteintech, 1 : 1000), anti-Hsp90 (H-114, Santa Cruz Biotechnology, 1 : 1000) and mouse anti-HuR (3A2, Santa Cruz Biotechnology, 1 : 200). Secondary antibodies were horseradish peroxidase-conjugated anti-rabbit and anti-mouse IgG antibodies (GE Healthcare, 1 : 5000).

### 4.6. siRNA-mediated knockdown of Mettl3, Elavl1 and m^6^A readers

Transient knockdown of target genes by siRNA was performed with Lipofectamine RNAi MAX (Invitrogen) and opti-MEM (Gibco by Thermo Fisher Scientific). The following siRNAs were used: siMettl3 (Dharmacon, ON-TARGETplus SMARTpool #L-049446), siElavl1 (Dharmacon, ON-TARGETplus SMARTpool #L-053812), siYthdf2 (Dharmacon, ON-TARGETplus SMARTpool #L-058271), siYthdc1 (Dharmacon, ON-TARGETplus SMARTpool #L-167076), siHnrnpa2b1 (Dharmacon, ON-TARGETplus SMARTpool #L-040194) and siHnrnpc (Dharmacon, ON-TARGETplus SMARTpool #L-044147).

### 4.7. Transfection

Seven MyoD mutants were generated as DNA inserts. Each insert DNA was amplified by PCR using KOD FX neo DNA polymerase (Toyobo Co., Ltd.) and ligated with the pCAGGS vector. A list of primers for cloning is shown in the electronic supplementary material, table S1. Plasmid transfection was performed using Lipofectamine 2000 (Invitrogen) following the manufacturer's instructions. The sequences of all mutants were confirmed by Sanger sequencing.

### 4.8. mRNA-seq

Total RNA was extracted from cells using Sepasol-RNA I Super G (Nacalai Tesque Inc.) and ethanol precipitation. Purified RNA samples underwent library construction using the NEBNext Ultra Directional RNA Library Prep Kit for Illumina (New England Biolabs, NEB #7420S). Library preparation and sequence analysis followed a previously described protocol [41].

Sequenced reads were mapped onto the mouse genome (mm9) using TopHat (version 2.0.8). Gene expression levels (fragments per kilobase of exon per million mapped sequence reads) were estimated using the Cuffdiff program in Cufflinks (version 2.0.1) using mapped reads and the software's default parameters [42].

### 4.9. m^6^A immunoprecipitation

m^6^A immunoprecipitation was performed according to a previously described protocol [31,43]. Total RNA was extracted from cells with Sepasol-RNA I Super G (Nacalai Tesque) and ethanol precipitation, and 348 µg was used for independent experiments. RNA was fragmented by mixing with 10 × fragmentation buffer (1 M ZnCl_2_, 1 M Tris–HCl pH 7.0 and RNase-free water) at 94°C for 5 min using a preheated thermal cycler. The fragmentation mix was ethanol precipitated by adding 1/10 volumes of 3 M sodium acetate, pH 5.2, glycogen (100 µg ml^−1^ final), and 2.5 volumes of 100% ethanol, mixing well, then incubating at –80°C overnight. Fragmented RNA was mixed with an anti-m^6^A antibody (Synaptic Systems, 202003, 12.5 µg) and 5 × immunoprecipitation buffer (0.05 M Tris–HCl, pH 7.4, 0.75 M NaCl, 0.5% Igepal CA-630, and RNase-free water). Ribonucleoside-vanadyl complexes (Sigma-Aldrich, R3380, 2 mM final) and RNasin Plus RNase inhibitor (Promega, N2611, 200–400 U, final) were mixed with immunoprecipitation reagents to prevent RNA degradation. Immunoprecipitation mixtures were incubated for 2 h at 4°C on a rotating wheel to allow the formation of antibody-RNA complexes. These complexes were mixed with Dynabeads Protein A (Novex by Life Technologies) and incubated for 2 h at 4°C on a rotating wheel. Immunoprecipitated RNA was eluted by competition with N^6^-methyladenosine, 5′-monophosphate sodium salt (m^6^A, Sigma-Aldrich, M2780, 6.7 mM final).

### 4.10. m^6^A-seq data analysis

Purified RNA fragments from m^6^A RNA immunoprecipitation underwent library construction using the NEBNext Ultra Directional RNA Library Prep Kit for Illumina (New England Biolabs, NEB #7420S). The m^6^A-seq library was sequenced on an Illumina HiSeq 1500 sequencing system. Sequence reads were aligned to the reference mouse genome (GRCm38) using HISAT2 software (version 2.0.4) [44]. The software deepTools2 (version 2.3.5) [45] was used to create the coverage tracks (BigWig file) of m^6^A-modified/input RNA on the mouse genome with the options: *bamCoverage* --*binSize* 1 --*normalizeTo1x2267226534* (the effective mouse genome size for 50 bp reads)’, and the m^6^A signal profiles on all coding sequences (taken from UCSC refSeq genes) with the options: *computeMatrix* -*m 2000* -*b 2000* -*a 2000* --*unscaled5prime 500* --*unscaled3prime 500* --*skipZeros* --*missingDataAsZero*.

### 4.11. Statistical analysis

Statistical significance in qRT-PCR data was evaluated using the two-sided Welch's *t*-test.

## Supplementary Material

Supplementary material

## Data accessibility

mRNA-seq and m^6^A-seq data were deposited into the DNA Data Bank of Japan database (accession no.: DRA005057). The other datasets supporting this article have been uploaded as part of the electronic supplementary material.

## References

[1] Niwa H, Burdon T, Chambers I, Smith A 1998 Self-renewal of pluripotent embryonic stem cells is mediated via activation of STAT3. Genes Dev. 12, 2048–2060. (doi:10.1101/gad.12.13.2048) 9649508 10.1101/gad.12.13.2048 PMC316954

[2] Singh SK, Kagalwala MN, Parker-Thornburg J, Adams H, Majumder S 2008 REST maintains self-renewal and pluripotency of embryonic stem cells. Nature 453, 223–227. (doi:10.1038/nature06863) 18362916 10.1038/nature06863 PMC2830094

[3] Thomson M, Liu SJ, Zou LN, Smith Z, Meissner A, Ramanathan S 2011 Pluripotency factors in embryonic stem cells regulate differentiation into germ layers. Cell 145, 875–889. (doi:10.1016/j.cell.2011.05.017) 21663792 10.1016/j.cell.2011.05.017 PMC5603300

[4] Munoz-Sanjuan I, Brivanlou AH 2002 Neural induction, the default model and embryonic stem cells. Nat. Rev. Neurosci. 3, 271–280. (doi:10.1038/nrn786) 11967557 10.1038/nrn786

[5] Abranches E, Silva M, Pradier L, Schulz H, Hummel O, Henrique D, Bekman E 2009 Neural differentiation of embryonic stem cells *in vitro*: a road map to neurogenesis in the embryo. PLoS ONE 4, e6286 (doi:101371/journal.pone.0006286) 19621087 10.1371/journal.pone.0006286 PMC2709448

[6] Wood WM, Etemad S, Yamamoto M, Goldhamer DJ 2013 MyoD-expressing progenitors are essential for skeletal myogenesis and satellite cell development. Dev. Biol. 384, 114–127. (doi:10.1016/j.ydbio.2013.09.012) 24055173 10.1016/j.ydbio.2013.09.012 PMC3838901

[7] Conerly ML, Yao Z, Zhong JW, Groudine M, Tapscott SJ 2016 Distinct activities of Myf5 and MyoD indicate separate roles in skeletal muscle lineage specification and differentiation. Dev. Cell 36, 375–385. (doi:10.1016/j.devcel.2016.01.021) 26906734 10.1016/j.devcel.2016.01.021 PMC4769793

[8] Harada A et al. 2012 Chd2 interacts with H3.3 to determine myogenic cell fate. EMBO J. 31, 2994–3007. (doi:10.1038/emboj.2012.136) 22569126 10.1038/emboj.2012.136 PMC3395093

[9] Zammit PS, Partridge TA, Yablonka-Reuveni Z 2006 The skeletal muscle satellite cell: the stem cell that came in from the cold. J. Histochem. Cytochem. 54, 1177–1191. (doi:101369/jhc.6R6995.2006) 16899758 10.1369/jhc.6R6995.2006

[10] Janssen I, Heymsfield SB, Wang ZM, Ross R 2000 Skeletal muscle mass and distribution in 468 men and women aged 18–88 yr. J. Appl. Physiol. 89, 81–88. 10904038 10.1152/jappl.2000.89.1.81

[11] Yaffe D, Saxel O 1977 Serial passaging and differentiation of myogenic cells isolated from dystrophic mouse muscle. Nature 270, 725–727. (doi:10.1038/270725a0) 563524 10.1038/270725a0

[12] Wang C, Liu W, Nie Y, Qaher M, Horton HE, Yue F, Asakura A, Kuang S 2017 Loss of MyoD Promotes Fate Transdifferentiation of myoblasts into brown adipocytes. EBioMedicine 16, 212–223. (doi:10.1016/j.ebiom.2017.01.015) 28117277 10.1016/j.ebiom.2017.01.015 PMC5474440

[13] Davis RL, Weintraub H, Lassar AB 1987 Expression of a single transfected cDNA converts fibroblasts to myoblasts. Cell 51, 987–1000. (doi:10.1016/0092-8674(87)90585-X) 3690668 10.1016/0092-8674(87)90585-x

[14] Qin RF, Mao TQ, Gu XM, Hu KJ, Liu YP, Chen JW, Nie X 2007 Regulation of skeletal muscle differentiation in fibroblasts by exogenous MyoD gene *in vitro* and *in vivo*. Mol. Cell. Biochem. 302, 233–239. (doi:10.1007/s11010-007-9446-1) 17415623 10.1007/s11010-007-9446-1

[15] Berkes CA, Bergstrom DA, Penn BH, Seaver KJ, Knoepfler PS, Tapscott SJ 2004 Pbx marks genes for activation by MyoD indicating a role for a homeodomain protein in establishing myogenic potential. Mol. Cell 14, 465–477. (doi:10.1016/S1097-2765(04)00260-6) 15149596 10.1016/s1097-2765(04)00260-6

[16] de la Serna IL, Ohkawa Y, Berkes CA, Bergstrom DA, Dacwag CS, Tapscott SJ, Imbalzano AN 2005 MyoD targets chromatin remodeling complexes to the myogenin locus prior to forming a stable DNA-bound complex. Mol. Cell. Biol. 25, 3997–4009. (doi:101128/MCB.25.10.3997-4009.2005) 15870273 10.1128/MCB.25.10.3997-4009.2005 PMC1087700

[17] Simone C, Forcales SV, Hill DA, Imbalzano AN, Latella L, Puri PL 2004 p38 pathway targets SWI-SNF chromatin-remodeling complex to muscle-specific loci. Nat. Genet. 36, 738–743. (doi:10.1038/ng1378) 15208625 10.1038/ng1378

[18] Wang X et al. 2014 N6-methyladenosine-dependent regulation of messenger RNA stability. Nature 505, 117–120. (doi:10.1038/nature12730) 24284625 10.1038/nature12730 PMC3877715

[19] Batista PJ et al. 2014 m(6)A RNA modification controls cell fate transition in mammalian embryonic stem cells. Cell Stem Cell 15, 707–719. (doi:10.1016/j.stem.2014.09.019) 25456834 10.1016/j.stem.2014.09.019 PMC4278749

[20] Bokar JA, Shambaugh ME, Polayes D, Matera AG, Rottman FM 1997 Purification and cDNA cloning of the AdoMet-binding subunit of the human mRNA (N6-adenosine)-methyltransferase. RNA 3, 1233–1247. 9409616 PMC1369564

[21] Wang Y, Li Y, Toth JI, Petroski MD, Zhang Z, Zhao JC 2014 N6-methyladenosine modification destabilizes developmental regulators in embryonic stem cells. Nat. Cell Biol. 16, 191–198. (doi:10.1038/ncb2902) 24394384 10.1038/ncb2902 PMC4640932

[22] Fustin JM et al. 2013 RNA-methylation-dependent RNA processing controls the speed of the circadian clock. Cell 155, 793–806. (doi:10.1016/j.cell.2013.10.026) 24209618 10.1016/j.cell.2013.10.026

[23] Meyer KD, Patil DP, Zhou J, Zinoviev A, Skabkin MA, Elemento O, Pestova TV, Qian SB, Jaffrey SR 2015 5′ UTR m(6)A promotes cap-independent translation. Cell 163, 999–1010. (doi:10.1016/j.cell.2015.10.012) 26593424 10.1016/j.cell.2015.10.012 PMC4695625

[24] Wang X et al. 2015 N(6)-methyladenosine modulates messenger RNA translation efficiency. Cell 161, 1388–1399. (doi:10.1016/j.cell.2015.05.014) 26046440 10.1016/j.cell.2015.05.014 PMC4825696

[25] Figueroa A, Cuadrado A, Fan J, Atasoy U, Muscat GE, Munoz-Canoves P, Gorospe M, Munoz A 2003 Role of HuR in skeletal myogenesis through coordinate regulation of muscle differentiation genes. Mol. Cel. Biol. 23, 4991–5004. (doi:10.1128/MCB.23.14.4991-5004.2003) 10.1128/MCB.23.14.4991-5004.2003 PMC162217 12832484

[26] Lee JE, Lee JY, Wilusz J, Tian B, Wilusz CJ 2010 Systematic analysis of cis-elements in unstable mRNAs demonstrates that CUGBP1 is a key regulator of mRNA decay in muscle cells. PLoS ONE 5, e11201 (doi:101371/journal.pone.0011201) 20574513 10.1371/journal.pone.0011201 PMC2888570

[27] van der Giessen K, Di-Marco S, Clair E, Gallouzi IE 2003 RNAi-mediated HuR depletion leads to the inhibition of muscle cell differentiation. J. Biol. Chem. 278, 47 119–47 128. (doi:10.1074/jbc.M308889200) 10.1074/jbc.M308889200 12944397

[28] Kitzmann M, Carnac G, Vandromme M, Primig M, Lamb NJ, Fernandez A 1998 The muscle regulatory factors MyoD and myf-5 undergo distinct cell cycle-specific expression in muscle cells. J. Cell Biol. 142, 1447–1459. (doi:10.1083/jcb.142.6.1447) 9744876 10.1083/jcb.142.6.1447 PMC2141770

[29] Cao Y et al. 2010 Genome-wide MyoD binding in skeletal muscle cells: a potential for broad cellular reprogramming. Dev. Cell 18, 662–674. (doi:10.1016/j.devcel.2010.02.014) 20412780 10.1016/j.devcel.2010.02.014 PMC2910615

[30] Cammas A et al 2014 Destabilization of nucleophosmin mRNA by the HuR/KSRP complex is required for muscle fibre formation. Nat. Commun. 5, 4190 (doi:10.1038/ncomms5190) 24969639 10.1038/ncomms5190 PMC4074165

[31] Dominissini D et al. 2012 Topology of the human and mouse m6A RNA methylomes revealed by m6A-seq. Nature 485, 201–206. (doi:10.1038/nature11112) 22575960 10.1038/nature11112

[32] Roundtree IA, He C 2016 Nuclear m(6)A reader YTHDC1 regulates mRNA splicing. Trends Genet. 32, 320–321. (doi:10.1016/j.tig.2016.03.006) 27050931 10.1016/j.tig.2016.03.006

[33] Xiao W et al. 2016 Nuclear m(6)A reader YTHDC1 regulates mRNA splicing. Mol. Cell 61, 507–519. (doi:10.1016/j.molcel.2016.01.012) 26876937 10.1016/j.molcel.2016.01.012

[34] Alarcon CR, Goodarzi H, Lee H, Liu X, Tavazoie S, Tavazoie SF 2015 HNRNPA2B1 is a mediator of m(6)A-dependent nuclear RNA processing events. Cell 162, 1299–1308. (doi:10.1016/j.cell.2015.08.011) 26321680 10.1016/j.cell.2015.08.011 PMC4673968

[35] Liu N, Dai Q, Zheng G, He C, Parisien M, Pan T 2015 N(6)-methyladenosine-dependent RNA structural switches regulate RNA-protein interactions. Nature 518, 560–564. (doi:10.1038/nature14234) 25719671 10.1038/nature14234 PMC4355918

[36] Zhou KI, Parisien M, Dai Q, Liu N, Diatchenko L, Sachleben JR, Pan T 2016 N(6)-Methyladenosine modification in a long noncoding RNA hairpin predisposes its conformation to protein binding. J. Mol. Biol. 428, 822–833. (doi:10.1016/j.jmb.2015.08.021) 26343757 10.1016/j.jmb.2015.08.021 PMC4779075

[37] Lin S, Choe J, Du P, Triboulet R, Gregory RI 2016 The m(6)A Methyltransferase METTL3 promotes translation in human cancer cells. Mol. Cell 62, 335–345. (doi:10.1016/j.molcel.2016.03.021) 27117702 10.1016/j.molcel.2016.03.021 PMC4860043

[38] Ke S et al. 2015 A majority of m6A residues are in the last exons, allowing the potential for 3′ UTR regulation. Genes Dev. 29, 2037–2053. (doi:101101/gad.269415.115) 26404942 10.1101/gad.269415.115 PMC4604345

[39] Meyer KD, Saletore Y, Zumbo P, Elemento O, Mason CE, Jaffrey SR 2012 Comprehensive analysis of mRNA methylation reveals enrichment in 3′ UTRs and near stop codons. Cell 149, 1635–1646. (doi:10.1016/j.cell.2012.05.003) 22608085 10.1016/j.cell.2012.05.003 PMC3383396

[40] Harada A, Maehara K, Sato Y, Konno D, Tachibana T, Kimura H, Ohkawa Y 2015 Incorporation of histone H3.1 suppresses the lineage potential of skeletal muscle. Nucleic Acids Res. 43, 775–786. (doi:10.1093/nar/gku1346) 25539924 10.1093/nar/gku1346 PMC4333396

[41] Odawara J, Harada A, Yoshimi T, Maehara K, Tachibana T, Okada S, Akashi K, Ohkawa Y 2011 The classification of mRNA expression levels by the phosphorylation state of RNAPII CTD based on a combined genome-wide approach. BMC Genomics 12, 516 (doi:101186/1471-2164-12-516) 22011111 10.1186/1471-2164-12-516 PMC3209707

[42] Trapnell C et al. 2012 Differential gene and transcript expression analysis of RNA-seq experiments with TopHat and Cufflinks. Nat. Protoc. 7, 562–578. (doi:10.1038/nprot.2012.016) 22383036 10.1038/nprot.2012.016 PMC3334321

[43] Dominissini D, Moshitch-Moshkovitz S, Amariglio N, Rechavi G 2015 Transcriptome-Wide Mapping of N(6)-Methyladenosine by m(6)A-Seq. Methods Enzymol. 560, 131–147. (doi:10.1016/bs.mie.2015.03.001) 26253969 10.1016/bs.mie.2015.03.001

[44] Kim D, Langmead B, Salzberg SL 2015 HISAT: a fast spliced aligner with low memory requirements. Nat. Methods 12, 357–360. (doi:10.1038/nmeth.3317) 25751142 10.1038/nmeth.3317 PMC4655817

[45] Ramirez F, Ryan DP, Gruning B, Bhardwaj V, Kilpert F, Richter AS, Heyne S, Dundar F, Manke T 2016 deepTools2: a next generation web server for deep-sequencing data analysis. Nucleic Acids Res. 44, W160–W165. (doi:10.1093/nar/gkw257) 27079975 10.1093/nar/gkw257 PMC4987876

