# Supplementary material for: The requirement of Mettl3-promoted MyoD mRNA maintenance in proliferative myoblasts for skeletal muscle differentiation
Source: Open Biol. 2017 Sep 6;7(9):170119. doi: 10.1098/rsob.170119 (PMC5627051; doi:10.1098/rsob.170119)
Supplement: Supplementary material [file rsob170119supp1.pdf]

Supplementary material

The requirement of Mettl3-promoted *MyoD* mRNA maintenance in proliferative myoblasts for skeletal muscle differentiation

Kensuke Kudou[1,2], Tetsuro Komatsu[1], Jumpei Nogami[1], Kazumitsu Maehara[1], Akihito Harada[1], Hiroshi Saeki[2], Eiji Oki[2], Yoshihiko Maehara[2], and Yasuyuki Ohkawa[1]

[1]Division of Transcriptomics, Medical Institute of Bioregulation, Kyushu University, JST-CREST, Fukuoka 812-8582, Japan

[2]Department of Surgery and Science, Graduate School of Medical Sciences, Kyushu University, Fukuoka 812-8582, Japan

Corresponding author: Yasuyuki Ohkawa (yohkawa@bioreg.kyushu-u.ac.jp)

Figure S1

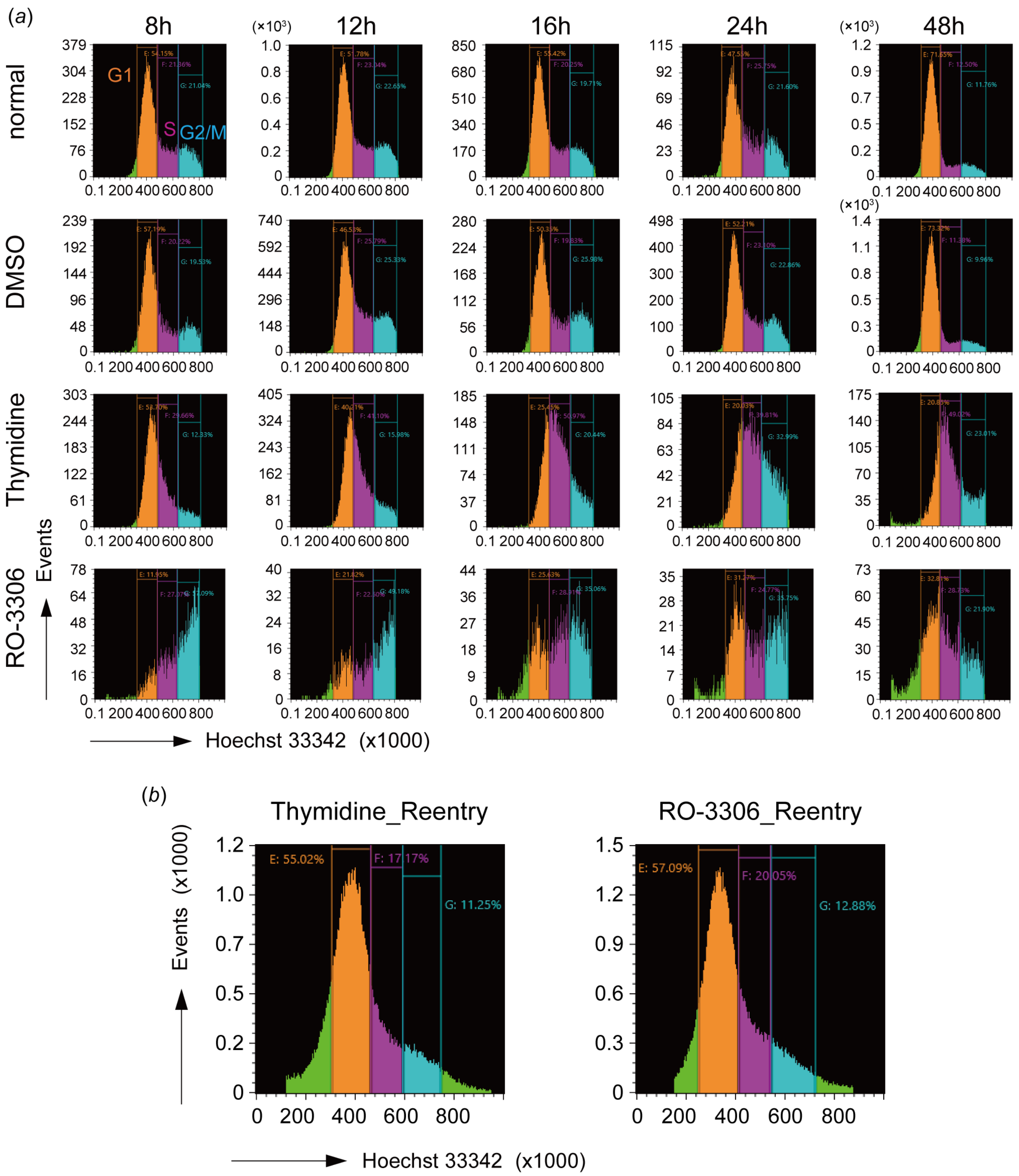

Figure S1. Cell treatments with thymidine and RO-3306. (*a*) Population changes of C2C12 cells in each cell cycle phase after incubation with thymidine, RO-3306, or DMSO. (*b*) Population of C2C12 cells treated with thymidine or RO-3306 in each cell cycle 24 h after re-entry into the cell cycle.

Figure S2

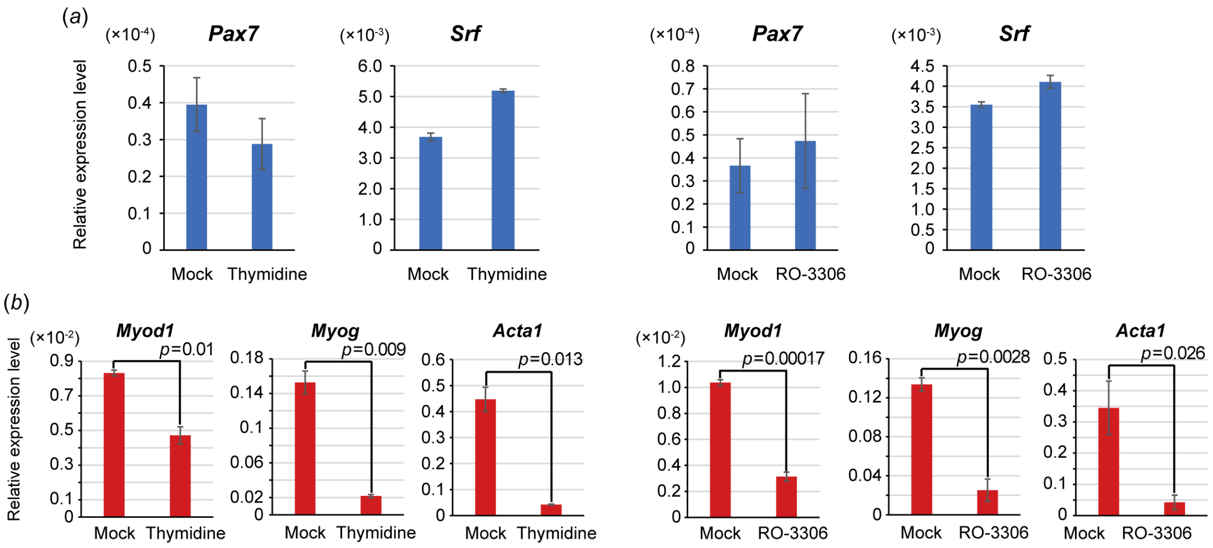

Figure S2. mRNA levels of skeletal muscle-specific factors upon cell cycle arrest. (*a*) qRT-PCR analysis of *Pax7* and *Srf* in undifferentiated C2C12 cells treated with thymidine (left panels) and RO-3306 (right panels) for 48 h. Data represent the mean ± SD (n=3). (*b*) qRT-PCR analysis of *Myod1*, *Myog*, and *Acta1* in C2C12 cells treated with thymidine (left panels) or RO-3306 (right panels) at 48 h after the induction of differentiation.

Figure S3

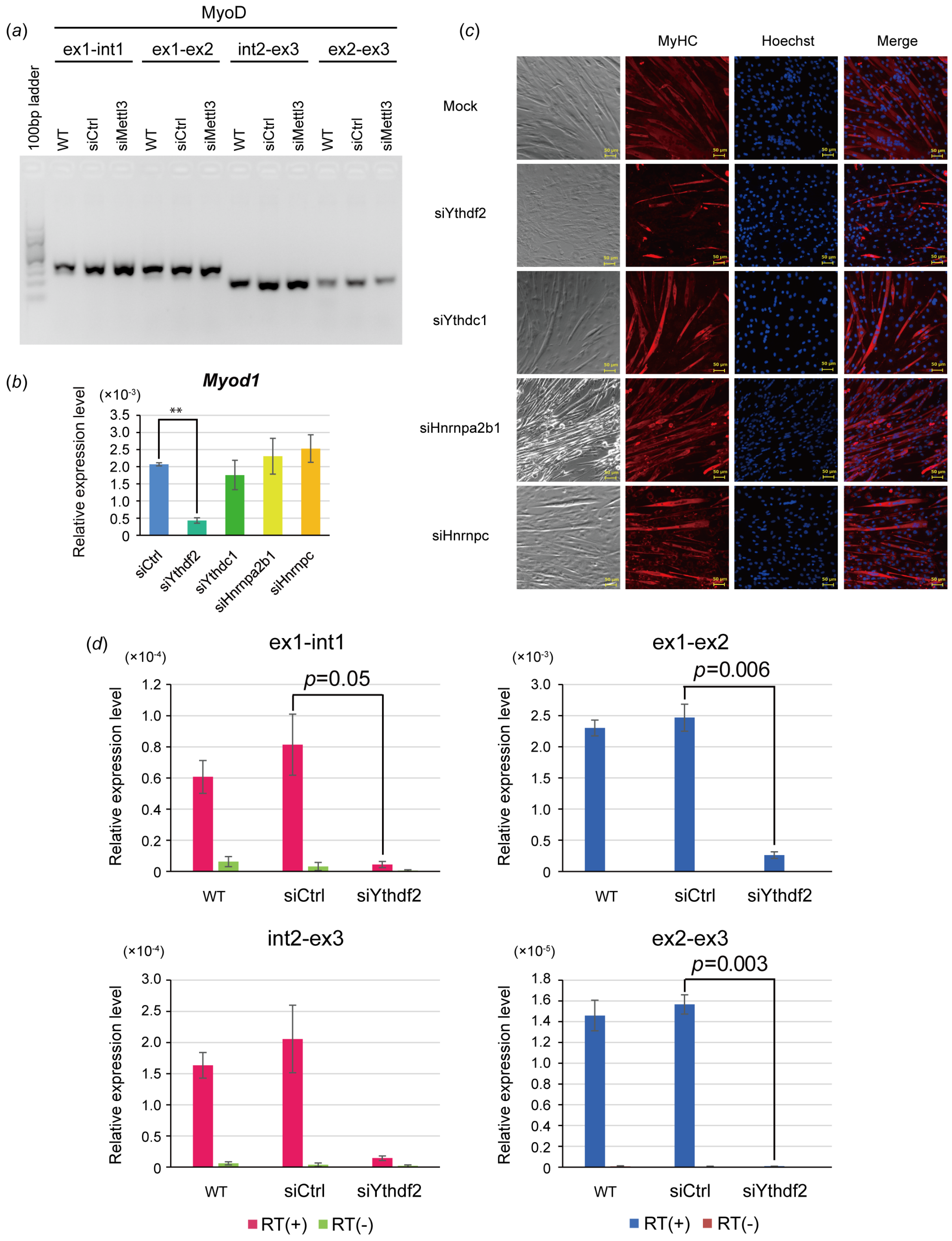

(a)
MyoD
ex1-int1
ex1-ex2
int2-ex3
ex2-ex3
100bp ladder
WT
siCtrl
siMettl3
(b)
Myod1
Relative expression level
(×10⁻³)
**
siCtrl
siYthdf2
siYthdc1
siHnrnpa2b1
siHnrnpc
(c)
MyHC
Hoechst
Merge
Mock
siYthdf2
siYthdc1
siHnrnpa2b1
siHnrnpc
(d)
ex1-int1
(×10⁻⁴)
p=0.05
ex1-ex2
(×10⁻³)
p=0.006
int2-ex3
(×10⁻⁴)
ex2-ex3
(×10⁻⁵)
p=0.003
Relative expression level
WT
siCtrl
siYthdf2
RT(+)
RT(-)

Figure S3. Knockdown of Ythdf2 leads to a decrease of both processed and unprocessed *MyoD* RNA. (*a*) Agarose gel electrophoresis of PCR products obtained using the four MyoD primer sets described in figure 3*a*. ex: exon, int: intron. (*b*) qRT-PCR analysis of *Myod1* in undifferentiated C2C12 cells after knockdown of Ythdf2, Ythdc1, Hnrnpa2b1, or Hnrnpc. Data are presented as the mean ± SD (n=3). (*c*) Morphology of C2C12 cells after knockdown of Ythdf2, Ythdc1, Hnrnpa2b1, or Hnrnpc. Cells were immunostained for anti-MyHC and Hoechst at 72 h after the induction of differentiation. (*d*) qRT-PCR analysis of *Myod1* in Ythdf2 knockdown C2C12 cells using the four primers described in figure 3*a*. To confirm the absence of DNA contamination, each sample was also analysed by qRT-PCR without reverse transcriptase. RT: reverse transcriptase. Error bars show ± SD (n=3). *P*-value versus control siRNA.

Figure S4

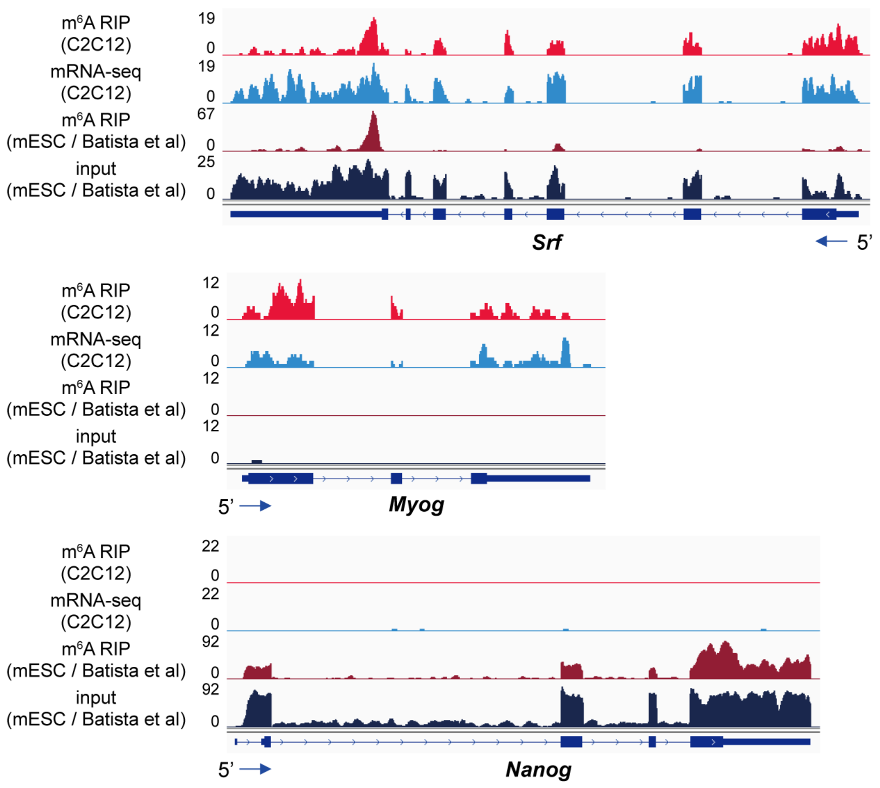

Figure S4. m[6]A-seq analysis using C2C12 and mouse embryonic stem cells. Integrative Genomics Viewer tracks displaying reads coverage of *Myog*, *Srf*, and *Nanog* in m[6]A-seq of total RNA and mRNA-seq data of C2C12 cells and mouse embryonic stem cells (mESC) (SRR1207291 (input), SRR1207292 (m[6]A RIP), Batista et al. 2014).

List of primers for qRT-PCR	
Myod1_F	CAACCCACAGAACCTTTGTCATTG
Myod1_R	GTCGAAACACGGGTCATCATAGAA
Gapdh_F	TTCCTACCCCCAATGTGTCCGTCG
Gapdh_R	GTGGGAGTTGCTGTTGAAGTCGCA
Eef1a1_F	CTCTGACTACCCTCCACTTGGTCG
Eef1a1_R	ATTAAGACTGGGGTGGCAGGTGTT
Myog_F	AAAGCCATCACTTCTGTAGCAGGG
Myog_R	TCTCTGGACTCCATCTTTCTCTCC
Acta1_F	TCCTCAGGACGACAATCGACAATC
Acta1_R	TTCCTTTCCACAGGGCTTTGTTTG
Mettl3_F	CTCTGGGCACTTGGATTTAAGGAA
Mettl3_R	AGCAACTTCTTCTCTAACTCAGGG
Elavl1_F	AGAACATGACCCAAGAGGAACTAC
Elavl1_R	GACACCTTAATGGTTTTGGACTGG
Srf_F	ATGAGTATTAGCTGACCCGATGGG
Srf_R	GGCAGACACAAAGCTAACCAGAGG
Pax7_F	TCTCGGGCCAGACAAAATTGCTGC
Pax7_R	ATGGGGTGGCTTAGAAACAGTGCC
Myod1_ex1_Fw	AGCAAAGTGAATGAGGCCTTCGAG
Myod1_int1_Rv	ATCTAGGTATAAGGGACACCCCCA
Myod1_ex2_Rv	TCACTGTAGTAGGCGGTGTCGTAG
Myod1_int2_Fw	ATTACTAACCTTCCACTCCCCTCA
Myod1_ex2_Fw	GCTACGACACCGCCTACTACAGTG
Myod1_ex3_Rv	CCTGTTCTGTGTCGCTTAGGGATG
Myod1_exon1_CDS_F	CCGCCTGAGCAAAGTGAATGAGGC
Myod1_exon1_CDS_R	CCTTCGATGTAGCGGATGGCGTTG
List of primers for cloning	
Myod1_full length_F	CGGAATTCCCCTACTACACTCCTATTGGCTTGAGGC
Myod1_full length_R	CGCAATTGCCCTTCTTCATTACCCAGCACTACCCAG
Myod1_5'UTR_deletion_F	CGGAATTCATGGAGCTTCTATCGCCGCCAC
Myod1_5'UTR_deletion_R	CGGAATTCGCCACTTTGTATAAATTAGCGTC
Myod1_Mut1_F	TGACAGGCCAGGCCAGGGAGGAGGGGTAGA
Myod1_Mut1_R	TCCCTGGCCTGGCCTGTCAGAGGTGTGGTG
Myod1_Mut2_F	GTAGAGGCCAGCCGGTGTGCATTCCAACCC
Myod1_Mut2_R	CGGCTGGCCTCTACCCCTCCTCCCTGTCCT
Myod1_6bp-del_F	CACACCTCTGACAGGCAGGGAGGAGGGGTA
Myod1_6bp-del_R	TACCCCTCCTCCCTGCCTGTCAGAGGTGTG
Myod1_3'UTR_deletion_F	CGGAATTCCCCTACTACACTCCTATTGGCTTGAGGC
Myod1_3'UTR_deletion_R	CGGAATTCTCAAAGCACCTGATAAATCGCATTGGGG
Myod1_CDS_F	CGTCTAGAATGGAGCTTCTATCGCCGCCACTCC
Myod1_CDS_R	CGGAATTCTCAAAGCACCTGATAAATCGCATTG

Table S1. List of primers used for RT-PCR and cloning.
